# Supplementary material for: Disturbed intramitochondrial phosphatidic acid transport impairs cellular stress signaling
Source: J Biol Chem. 2021 Jan 23;296:100335. doi: 10.1016/j.jbc.2021.100335 (PMC7949116; doi:10.1016/j.jbc.2021.100335)
Supplement: Supplementary file 1 — Figures S1 to S3; Tables S5 & S6 [file mmc1.pdf]

Supporting information

Disturbed intramitochondrial phosphatidic acid transport impairs cellular stress signaling

Akinori Eiyama[1], Mari J Aaltonen[2,†], Hendrik Nolte[1], Takashi Tatsuta[1] and Thomas Langer[1,2,*]

[1]Max-Planck-Institute for Biology of Ageing, Cologne, Germany

[2]Cologne Excellence Cluster on Cellular Stress Responses in Aging-Associated Diseases (CECAD), University of Cologne, Cologne, Germany

[†]Present address: Montreal Neurological Institute, McGill University, Montreal, QC, Canada

[*]Corresponding author: Thomas Langer

Email: TLanger@age.mpg.de

Figure legends

Figure S1. Phospholipidome of *ups1*Δ and wild-type (WT) cells

(A) Scatter plot of whole cell phospholipidome in *ups1*Δ cells against wild-type (WT) cells. Dashed line represents $p = 0.05$, the colors of circles represent lipid class, and the areas of circles are proportional to the relative abundance of individual lipid species in WT.

(B) Scatter plot of ER-enriched microsome phospholipidome in *ups1*Δ cells against WT cells. Dashed line represents $p = 0.05$, the colors of circles represent lipid class, and the areas of circles are proportional to the relative abundance of individual lipid species in WT.

(C) WT and *ups1*Δ cells expressing *4xUPRE-GFP* grown to log phase in SCGal medium were subjected to western blotting. Pgk1 and ponceau staining were monitored as a loading control.

(D) GFP in (C) was quantified. GFP signals were normalized to Pgk1 and expressed relative to WT cells (set as one). Data represent mean ± SD (n = 3).

(E) WT, *tam41*Δ and *pgs1*Δ cells expressing *4xUPRE-GFP* grown to log phase in SCD medium were subjected to western blotting. Pgk1 and ponceau staining were monitored as a loading control.

(F) GFP in (E) was quantified. GFP signals were normalized to Pgk1 and expressed relative to WT cells (set as one). Data represent mean ± SD (n = 3).

(G) WT, *ups1*Δ and *crd1*Δ cells expressing *4xUPRE-GFP* grown to log phase in SCD medium were subjected to western blotting.

(H) GFP in (G) was quantified. GFP signals were normalized to Pgk1 and expressed relative to WT cells (set as one). Data represent mean ± SD (n = 3).

Figure S2. RNA-seq data suggest suppression of the UPR and induction of autophagy in *ups1*Δ cells

(A) Bar graph of relative abundance of canonical UPR regulated genes in *ups1*Δ cells against wild-type (WT) cells. Total RNA was extracted from WT and *ups1*Δ cells grown to log phase in SCD medium. RNA samples were subjected to RNA-seq analysis. (n = 3). ***P* < 0.01.

(B) Bar graph of relative abundance of significantly changed core *ATG* genes in *ups1*Δ cells against WT cells. ***P* < 0.01.

(C) Scatter plot of gene abundance in WT+tunicamycin/WT against *ups1*Δ+tunicamycin/ *ups1*Δ. Genes changed significantly ($P < 0.05$) by treatment with tunicamycin in WT and *ups1*Δ cells are plotted. Orange highlight indicates genes involved in ribosome biogenesis (RiBi genes).

Figure S3. Alterations in the phospholipid composition of cell membranes do not significantly affect TORC1-Sch9 signaling

(A) Cardiolipin (CL) levels of wild-type (WT) and *crd1*Δ cells grown to log phase in SCD medium. Data represent mean ± SD (n = 3).

(B) WT, *ups1*Δ, *ups2*Δ and *crd1*Δ cells expressing Sch9-6HA were grown to log phase in SCD medium. For the analysis of Sch9 phosphorylation, lysates were treated with NTCB and subjected to western blotting.

(C) Phosphorylated Sch9-6HA ratio in (B) was quantified. Phosphorylated Sch9-6HA signals were divided with total Sch9-6HA signals. Data represent mean ± SD (n = 3). **P* < 0.05. N.S., not significant.

(D) WT cells expressing Sch9-6HA were grown to log phase in SCD medium with or without 1 mM choline. For the analysis of Sch9 phosphorylation, lysates were treated with NTCB and subjected to western blotting.

(E) Phosphorylated Sch9-6HA ratio in (D) was quantified as described in (F). Data represent mean ± SD (n = 3). N.S., not significant.

(F) WT, *nem1*Δ, and *spo7*Δ cells expressing Sch9-6HA were grown to log phase in SCD medium. For the analysis of Sch9 phosphorylation, lysates were treated with NTCB and subjected to western blotting.
(G) Phosphorylated Sch9-6HA ratio in (F) was quantified. Phosphorylated Sch9-6HA signals were divided with total Sch9-6HA signals. Data represent mean ± SD (n = 3). N.S., not significant.

Fig. S1

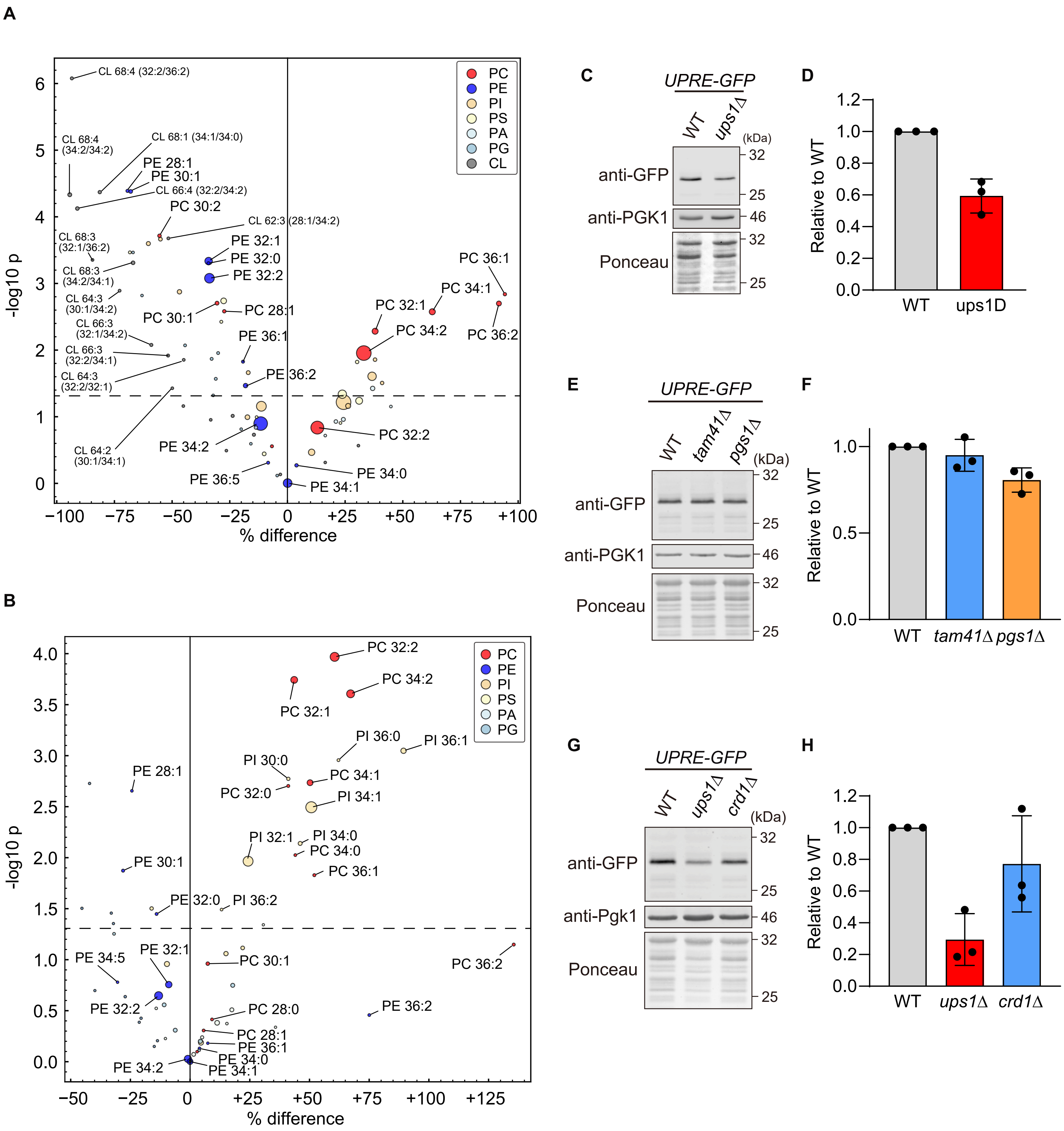
A
-log10 p
% difference
PC
PE
PI
PS
PA
PG
CL
CL 68:4 (32:2/36:2)
CL 68:4
(34:2/34:2)
CL 68:1 (34:1/34:0)
PE 28:1
PE 30:1
CL 66:4 (32:2/34:2)
PC 30:2
CL 62:3 (28:1/34:2)
CL 68:3
(32:1/36:2)
PE 32:1
PE 32:0
PE 32:2
CL 68:3
(34:2/34:1)
CL 64:3
(30:1/34:2)
PC 30:1
PC 28:1
CL 66:3
(32:1/34:2)
CL 66:3
(32:2/34:1)
CL 64:3
(32:2/32:1)
PE 36:1
PE 36:2
CL 64:2
(30:1/34:1)
PE 34:2
PE 36:5
PE 34:1
PE 34:0
PC 32:2
PC 32:1
PC 34:2
PC 34:1
PC 36:1
PC 36:2
B
-log10 p
% difference
PC 32:2
PC 34:2
PC 32:1
PI 36:0
PI 36:1
PI 30:0
PE 28:1
PC 34:1
PC 32:0
PI 34:1
PI 32:1
PI 34:0
PC 34:0
PC 36:1
PE 30:1
PE 32:0
PI 36:2
PE 34:5
PE 32:1
PC 30:1
PC 36:2
PE 32:2
PC 28:0
PE 36:2
PC 28:1
PE 36:1
PE 34:0
PE 34:2
PE 34:1
C
UPRE-GFP
WT
ups1Δ
(kDa)
anti-GFP
anti-PGK1
Ponceau
D
Relative to WT
WT
ups1D
E
UPRE-GFP
WT
tam41Δ
pgs1Δ
(kDa)
anti-GFP
anti-PGK1
Ponceau
F
Relative to WT
WT
tam41Δ
pgs1Δ
G
UPRE-GFP
WT
ups1Δ
crd1Δ
(kDa)
anti-GFP
anti-Pgk1
Ponceau
H
Relative to WT
WT
ups1Δ
crd1Δ

Fig. S2

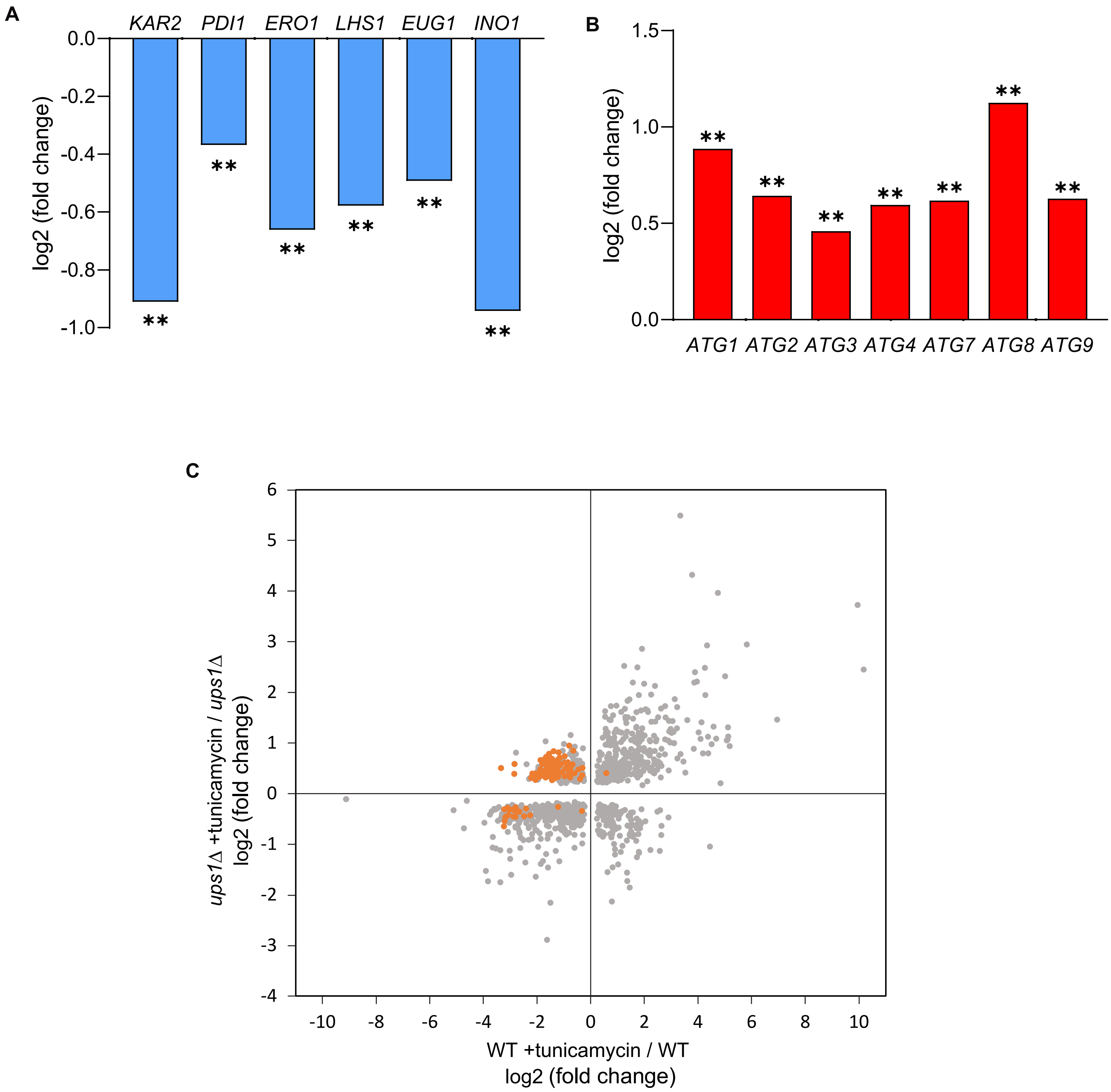

A
KAR2
PDI1
ERO1
LHS1
EUG1
INO1
0.0
-0.2
-0.4
-0.6
-0.8
-1.0
log2 (fold change)
**
B
1.5
1.0
0.5
0.0
log2 (fold change)
ATG1
ATG2
ATG3
ATG4
ATG7
ATG8
ATG9
C
6
5
4
3
2
1
0
-1
-2
-3
-4
-10
-8
-6
-4
-2
0
2
4
6
8
10
ups1Δ +tunicamycin / ups1Δ
log2 (fold change)
WT +tunicamycin / WT
log2 (fold change)

Fig. S3

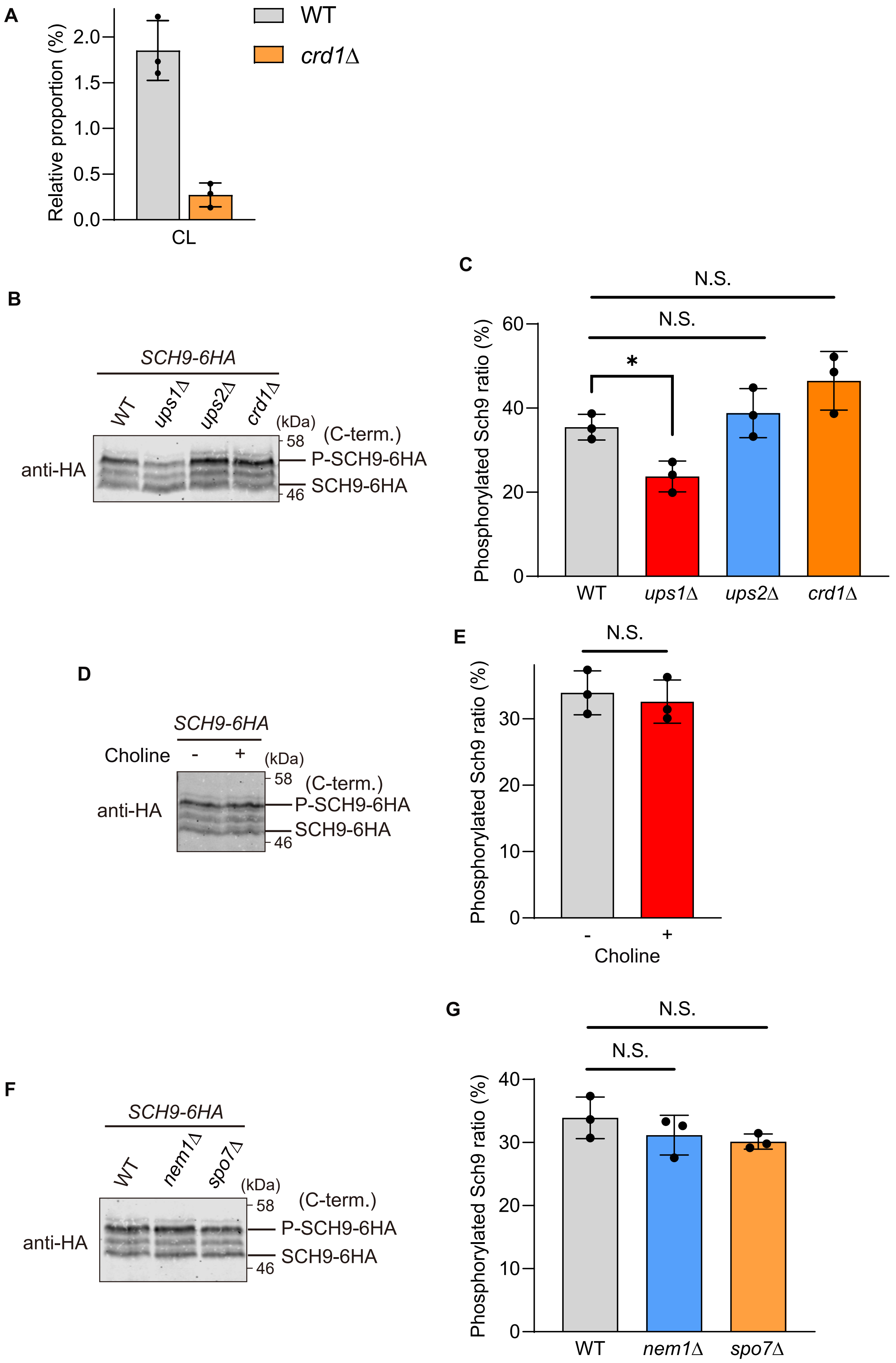
A
WT
crd1Δ
Relative proportion (%)
2.0
1.5
1.0
0.5
0.0
CL
B
SCH9-6HA
WT
ups1Δ
ups2Δ
crd1Δ
(kDa)
58
46
(C-term.)
anti-HA
P-SCH9-6HA
SCH9-6HA
C
N.S.
N.S.
*
Phosphorylated Sch9 ratio (%)
60
40
20
0
WT
ups1Δ
ups2Δ
crd1Δ
D
SCH9-6HA
Choline
-
+
(kDa)
58
46
(C-term.)
anti-HA
P-SCH9-6HA
SCH9-6HA
E
N.S.
Phosphorylated Sch9 ratio (%)
30
20
10
0
-
+
Choline
F
SCH9-6HA
WT
nem1Δ
spo7Δ
(kDa)
58
46
(C-term.)
anti-HA
P-SCH9-6HA
SCH9-6HA
G
N.S.
N.S.
Phosphorylated Sch9 ratio (%)
40
30
20
10
0
WT
nem1Δ
spo7Δ

Table S1. RNA-seq data.

Table S2. Proteomics data.

Table S3. RNA-seq GO term DAVID.

Table S4. Proteomics 1D enrichment.

Table S5. Strain list

strain name	strain description	background	Mating type	genotype	source
CG214	wild-type	S288c	a	*his3Δ1leu2Δ0 met15Δ0 lys2Δ0 ura3Δ0*	Osman et al., 2009
PD49	*ups1Δ*	S288c	α	*his3Δ1leu2Δ0 ura3Δ0 ups1Δ::hphNT1*	Osman et al., 2009
MA318	*UPRE-GFP-URA3*	W303	a	*ade2Δ1 leu2Δ3 his3Δ11, 15 trp1Δ1 ura3Δ1 can1Δ100 ura3Δ::4xUPRE-GFP-URA3*	This study
MA657	*UPRE-GFP-URA3 ups1Δ*	W303	a	*ade2Δ1 leu2–3 his3Δ11,15 trp1Δ1 ura3Δ1 can1Δ100 ura3Δ::4xUPRE-GFP-URA3 ups1Δ::hphNT1*	This study
AE47	*IRE1(ΔIII) -3xHA-GFP*	S288c	a	*his3Δ1leu2Δ0 met15Δ0 lys2Δ0 ura3Δ0 IRE1::IRE1(ΔIII) -3xHA-GFP*	Halbleib et al., 2017
AE207	*IRE1-3xHA-GFP(ΔIII) UPRE-GFP-URA3*	S288c	a	*his3Δ1leu2Δ0 met15Δ0 lys2Δ0 ura3Δ0 IRE1::IRE1-3xHA-GFP(ΔIII) ura3Δ::4xUPRE-GFP-URA3*	This study
AE223	*IRE1-3xHA-GFP(ΔIII) UPRE-GFP-URA3 ups1Δ*	S288c	a	*his3Δ1leu2Δ0 met15Δ0 lys2Δ0 ura3Δ0 IRE1::IRE1-3xHA-GFP(ΔIII) ura3Δ::4xUPRE-GFP-URA3 ups1Δ::hphNT1*	This study
AE4	wild-type +pRS315-IRE1	S288c	a	*his3Δ1leu2Δ0 met15Δ0 lys2Δ0 ura3Δ0 +pRS315-IRE1*	This study
AE5	*ups1Δ* +pRS315-IRE1	S288c	α	*his3Δ1leu2Δ0 ura3Δ0 Δups1::hphNT1 +pRS315-IRE1*	This study
AE23	wild-type +pRS315	S288c	a	*his3Δ1leu2Δ0 met15Δ0 lys2Δ0 ura3Δ0 +pRS315*	This study
AE24	*ups1Δ* +pRS315	S288c	α	*his3Δ1leu2Δ0 ura3Δ0 Δups1::hphNT1 +pRS315*	This study
AE321	*hac1Δ* +pRS315	S288c	a	*his3Δ1leu2Δ0 met15Δ0 lys2Δ0 ura3Δ0 hac1Δ::kanMX6 +pRS315*	This study
AE322	*hac1Δ* +pRS315-IRE1	S288c	a	*his3Δ1leu2Δ0 met15Δ0 lys2Δ0 ura3Δ0 hac1Δ::kanMX6 +pRS315-IRE1*	This study
AE323	*ups1Δ hac1Δ* +pRS315	S288c	α	*his3Δ1leu2Δ0 ura3Δ0 ups1Δ::hphNT1 hac1Δ::kanMX6 +pRS315*	This study
AE324	*ups1Δ hac1Δ* +pRS315-IRE1	S288c	α	*his3Δ1leu2Δ0 ura3Δ0 ups1Δ::hphNT1 hac1Δ::kanMX6 +pRS315-IRE1*	This study
AE326	*ire1Δ* +pRS315-IRE1	S288c	a	*his3Δ1leu2Δ0 met15Δ0 lys2Δ0 ura3Δ0 ire1Δ::kanMX6 +pRS315-IRE1*	This study
AE75	*UPRE-GFP-URA3* +pRS315	W303	a	*ade2Δ1 leu2Δ3 his3Δ11, 15 trp1Δ1 ura3Δ1 can1Δ100 ura3Δ::4xUPRE-GFP-URA3 +pRS315*	This study

AE76	*UPRE-GFP-URA3* +pRS315-IRE1	W303	a	*ade2Δ1 leu2Δ3 his3Δ11, 15 trp1Δ1 ura3Δ1 can1Δ100 ura3Δ::4xUPRE-GFP-URA3 +pRS315-IRE1*	This study
AE77	*UPRE-GFP-URA3 ups1Δ* +pRS315	W303	a	*ade2Δ1 leu2–3 his3Δ11,15 trp1Δ1 ura3Δ1 can1Δ100 ura3Δ::4xUPRE-GFP-URA3 ups1Δ::hphNT1 +pRS315*	This study
AE78	*UPRE-GFP-URA3 ups1Δ* +pRS315-IRE1	W303	a	*ade2Δ1 leu2–3 his3Δ11,15 trp1Δ1 ura3Δ1 can1Δ100 ura3Δ::4xUPRE-GFP-URA3 ups1Δ::hphNT1 +pRS315-IRE1*	This study
AE239	*cho2Δ*	S288c	a	*his3Δ1leu2Δ0 met15Δ0 lys2Δ0 ura3Δ0 cho2Δ::hphNT1*	This study
AE240	*ups1Δ cho2Δ*	S288c	α	*his3Δ1leu2Δ0 ura3Δ0 ups1Δ::hphNT1 cho2Δ::kanMX6*	This study
AE241	*opi3Δ*	S288c	a	*his3Δ1leu2Δ0 met15Δ0 lys2Δ0 ura3Δ0 opi3Δ::hphNT1*	This study
AE243	*ups1Δ opi3Δ*	S288c	α	*his3Δ1leu2Δ0 ura3Δ0 ups1Δ::hphNT1 opi3Δ::kanMX6*	This study
AE149	*UPRE-GFP-URA3 cho2Δ*	W303	a	*ade2Δ1 leu2Δ3 his3Δ11, 15 trp1Δ1 ura3Δ1 can1Δ100 ura3Δ::4xUPRE-GFP-URA3 cho2Δ::kanMX6*	This study
AE150	*UPRE-GFP-URA3 ups1Δ cho2Δ*	W303	a	*ade2Δ1 leu2–3 his3Δ11,15 trp1Δ1 ura3Δ1 can1Δ100 ura3Δ::4xUPRE-GFP-URA3 ups1Δ::hphNT1 cho2Δ::kanMX6*	This study
AE152	*UPRE-GFP-URA3 opi3Δ*	W303	a	*ade2Δ1 leu2Δ3 his3Δ11, 15 trp1Δ1 ura3Δ1 can1Δ100 ura3Δ::4xUPRE-GFP-URA3 cho2Δ::kanMX6*	This study
AE154	*UPRE-GFP-URA3 ups1Δ opi3Δ*	W303	a	*ade2Δ1 leu2–3 his3Δ11,15 trp1Δ1 ura3Δ1 can1Δ100 ura3Δ::4xUPRE-GFP-URA3 ups1Δ::hphNT1 opi3Δ::kanMX6*	This study
AE257	*ire1Δ*	S288c	a	*his3Δ1leu2Δ0 met15Δ0 lys2Δ0 ura3Δ0 ire1Δ::kanMX6*	This study
AE258	*ups1Δ ire1Δ*	S288c	α	*his3Δ1leu2Δ0 ura3Δ0 ups1Δ::hphNT1 ire1Δ::kanMX6*	This study
AE317	*hac1Δ*	S288c	a	*his3Δ1leu2Δ0 met15Δ0 lys2Δ0 ura3Δ0 hac1Δ::kanMX6*	This study
AE319	*ups1Δ hac1Δ*	S288c	α	*his3Δ1leu2Δ0 ura3Δ0 ups1Δ::hphNT1 hac1Δ::kanMX6*	This study
AE335	*SCH9-6HA*	S288c	a	*his3Δ1leu2Δ0 met15Δ0 lys2Δ0 ura3Δ0 SCH9-6HA::natNT2*	This study
AE337	*ups1Δ SCH9-6HA*	S288c	α	*his3Δ1leu2Δ0 ura3Δ0 ups1Δ::hphNT1 SCH9-6HA::natNT2*	This study
AE344	*npr2Δ*	S288c	a	*his3Δ1leu2Δ0 met15Δ0 lys2Δ0 ura3Δ0 npr2Δ::natNT2*	This study

AE346	*ups1Δ npr2Δ*	S288c	α	*his3Δ1leu2Δ0 ura3Δ0 ups1Δ::hphNT1 npr2Δ::natNT2*	This study
AE348	*npr3Δ*	S288c	a	*his3Δ1leu2Δ0 met15Δ0 lys2Δ0 ura3Δ0 npr3Δ::natNT2*	This study
AE349	*ups1Δ npr3Δ*	S288c	α	*his3Δ1leu2Δ0 ura3Δ0 ups1Δ::hphNT1 npr3Δ::natNT2*	This study
AE363	wild-type +pRS416	S288c	a	*his3Δ1leu2Δ0 met15Δ0 lys2Δ0 ura3Δ0 +pRS416*	This study
AE364	wild-type +pRS416-SCH9	S288c	a	*his3Δ1leu2Δ0 met15Δ0 lys2Δ0 ura3Δ0 +pRS416-SCH9*	This study
AE365	wild-type +pRS416-SCH9(T723A, S726A, T737A, S758A, S765A)	S288c	a	*his3Δ1leu2Δ0 met15Δ0 lys2Δ0 ura3Δ0 +pRS416-SCH9(T723A, S726A, T737A, S758A, S765A)*	This study
AE366	wild-type +pRS416-SCH9(T723D, S726D, T737E, S758E, S765E)	S288c	a	*his3Δ1leu2Δ0 met15Δ0 lys2Δ0 ura3Δ0 +pRS416-SCH9(T723D, S726D, T737E, S758E, S765E)*	This study
AE367	*ups1Δ* +pRS416	S288c	α	*his3Δ1leu2Δ0 ura3Δ0 ups1Δ::hphNT1 +pRS416*	This study
AE368	*ups1Δ* +pRS416-SCH9	S288c	α	*his3Δ1leu2Δ0 ura3Δ0 ups1Δ::hphNT1 +pRS416-SCH9*	This study
AE369	*ups1Δ* +pRS416-SCH9(T723A, S726A, T737A, S758A, S765A)	S288c	α	*his3Δ1leu2Δ0 ura3Δ0 ups1Δ::hphNT1 +pRS416-SCH9(T723A, S726A, T737A, S758A, S765A)*	This study
AE370	*ups1Δ* +pRS416-SCH9(T723D, S726D, T737E, S758E, S765E)	S288c	α	*his3Δ1leu2Δ0 ura3Δ0 ups1Δ::hphNT1 +pRS416-SCH9(T723D, S726D, T737E, S758E, S765E)*	This study
AE399	*UPRE-GFP-URA3* +pRS416	W303	a	*ade2Δ1 leu2Δ3 his3Δ11, 15 trp1Δ1 ura3Δ1 can1Δ100 ura3Δ::4xUPRE-GFP-URA3 +pRS416*	This study
AE403	*UPRE-GFP-URA3 ups1Δ* +pRS416	W303	a	*ade2Δ1 leu2Δ3 his3Δ11, 15 trp1Δ1 ura3Δ1 can1Δ100 ura3Δ::4xUPRE-GFP-URA3 ups1Δ::hphNT1+pRS416*	This study
AE404	*UPRE-GFP-URA3 ups1Δ* +pRS416-SCH9	W303	a	*ade2Δ1 leu2Δ3 his3Δ11, 15 trp1Δ1 ura3Δ1 can1Δ100 ura3Δ::4xUPRE-GFP-URA3 ups1Δ::hphNT1+pRS416-SCH9*	This study
AE405	*UPRE-GFP-URA3 ups1Δ* +pRS416-SCH9(T723A, S726A, T737A, S758A, S765A)	W303	a	*ade2Δ1 leu2Δ3 his3Δ11, 15 trp1Δ1 ura3Δ1 can1Δ100 ura3Δ::4xUPRE-GFP-URA3 ups1Δ::hphNT1 +pRS416-SCH9(T723A, S726A, T737A, S758A, S765A)*	This study
AE406	*UPRE-GFP-URA3 ups1Δ* +pRS416-SCH9(T723D, S726D, T737E, S758E, S765E)	W303	a	*ade2Δ1 leu2Δ3 his3Δ11, 15 trp1Δ1 ura3Δ1 can1Δ100 ura3Δ::4xUPRE-GFP-URA3 ups1Δ::hphNT1 +pRS416-SCH9(T723D, S726D, T737E, S758E, S765E)*	This study
AE352	*ups2Δ SCH9-6HA*	S288c	a	*his3Δ1leu2Δ0 ura3Δ0 ups2Δ::natNT2 SCH9-6HA::hphNT1*	This study
AE354	*crd1Δ SCH9-6HA*	S288c	a	*his3Δ1leu2Δ0 ura3Δ0 crd1Δ::kanMX6 SCH9-6HA::natNT2*	This study

AE359	*nem1Δ SCH9-6HA*	S288c	a	*his3Δ1leu2Δ0 ura3Δ0 nem1Δ::hphNT1 SCH9-6HA::natNT2*	This study
AE360	*spo7Δ SCH9-6HA*	S288c	a	*his3Δ1leu2Δ0 ura3Δ0 spo7Δ::hphNT1 SCH9-6HA::natNT2*	This study
AE103	*UPRE-GFP-URA3 tam41Δ*	W303	a	*ade2Δ1 leu2–3 his3Δ11,15 trp1Δ1 ura3Δ1 can1Δ100 ura3Δ::4xUPRE-GFP-URA3 tam41Δ::kanMX6*	This study
AE105	*UPRE-GFP-URA3 pgs1Δ*	W303	a	*ade2Δ1 leu2–3 his3Δ11,15 trp1Δ1 ura3Δ1 can1Δ100 ura3Δ::4xUPRE-GFP-URA3 tam41Δ::kanMX6*	This study
AE316	*UPRE-GFP-URA3 crd1Δ*	W303	a	*ade2Δ1 leu2Δ3 his3Δ11, 15 trp1Δ1 ura3Δ1 can1Δ100 ura3Δ::4xUPRE-GFP-URA3 crd1Δ::kanMX6*	This study

Osman, C., Haag, M., Potting, C., Rodenfels, J., Dip, P. V., Wieland, F. T., Brugger, B., Westermann, B., and Langer, T. (2009) The genetic interactome of prohibitins: coordinated control of cardiolipin and phosphatidylethanolamine by conserved regulators in mitochondria. J Cell Biol 184, 583-596

Halbleib, K., Pesek, K., Covino, R., Hofbauer, H. F., Wunnicke, D., Hanelt, I., Hummer, G., and Ernst, R. (2017) Activation of the Unfolded Protein Response by Lipid Bilayer Stress. Mol Cell 67, 673-684 e678

Table S6. Plasmid list

name	source
pRS315	Sikorsk et al., 1989
pRS315-IRE1	Velázquez et al., 2016
pRS416	Sikorsk et al., 1989
pRS416-SCH9	Urban et al., 2007
pRS416-SCH9(T723A, S726A, T737A, S758A, S765A)	Urban et al., 2007
pES416-SCH9(T723D, S726D, T737E, S758E, S765E)	Urban et al., 2007

Sikorski, R. S., and Hieter, P. (1989) A system of shuttle vectors and yeast host strains designed for efficient manipulation of DNA in Saccharomyces cerevisiae. *Genetics* 122, 19-27

Velázquez, A. P., Tatsuta, T., Ghillebert, R., Drescher, I., and Graef, M. (2016) Lipid droplet–mediated ER homeostasis regulates autophagy and cell survival during starvation. *J Cell Biol* 212, 621-631

Urban, J., Soulard, A., Huber, A., Lippman, S., Mukhopadhyay, D., Deloche, O., Wanke, V., Anrather, D., Ammerer, G., Riezman, H., Broach, J. R., De Virgilio, C., Hall, M. N., and Loewith, R. (2007) Sch9 is a major target of TORC1 in Saccharomyces cerevisiae. *Mol Cell* 26, 663-674
